# Heart Rate Variability and Coronary Artery Bypass Grafting: A Systematic Review

**DOI:** 10.31083/j.rcm2501036

**Published:** 2024-01-22

**Authors:** Patrycja S. Matusik, Omar Alomar, Maryam Rafaqat Hussain, Muhammad Akrmah, Paweł T. Matusik, Daniel M. Chen, Muhammed Alomar, Phyllis K. Stein

**Affiliations:** ^1^Chair of Radiology, Jagiellonian University Medical College and University Hospital, 30-688 Kraków, Poland; ^2^Heart Rate Variability Laboratory, Cardiovascular Division, Department of Medicine, Washington University School of Medicine in St. Louis, Saint Louis, MO 63130, USA; ^3^Icahn School of Medicine at Mount Sinai, New York, NY 10029, USA; ^4^Department of Pathology, Brigham and Women's Hospital, Boston, MA 02215, USA; ^5^Department of Electrocardiology, Institute of Cardiology, Faculty of Medicine, Jagiellonian University Medical College, 31-202 Kraków, Poland; ^6^Department of Electrocardiology, The John Paul II Hospital, 31-202 Kraków, Poland; ^7^Feinberg School of Medicine, Northwestern University, Chicago, IL 60611, USA

**Keywords:** heart rate variability, coronary artery bypass grafting surgery, mortality, atrial fibrillation, rehabilitation

## Abstract

**Background:**

Coronary artery bypass grafting (CABG) is a well-established surgical procedure used to treat significant coronary artery disease. Nevertheless, unfavorable cardiovascular events and complications, including cardiac arrhythmias may be observed in patients after CABG. Previous studies have revealed a relationship between risk of cardiac arrhythmias and abnormal heart rate variability (HRV), which reflects adverse alterations in cardiac autonomic functioning, that may occur in patients after a CABG procedure. The aim of this article was to provide a systematic review of the major research findings in this area.

**Methods:**

A literature search was carried out using PubMed, Cochrane, and Embase databases and relevant articles, published in English, were analyzed in detail.

**Results:**

Studies performed so far have shown time depending changes in HRV after CABG. Time and frequency domain HRV decrease acutely after CABG but recover almost completely to pre-operative values by 6 months after surgery. Some preoperative clinical states such as: heart failure, type 2 diabetes mellitus and depression adversely affect post-CABG HRV. Finally, post-CABG cardiac rehabilitation appears to improve exercise capacity and speed up recovery of HRV.

**Conclusions:**

Generally, traditional time and frequency domain HRV parameters fail to predict complications post-CABG. Altered non-linear measures of HRV may identify subgroups of subjects at increased risk of potential complications, including atrial fibrillation post-CABG. However, data available currently does not appear to unequivocally support the hypothesis that early HRV assessment in post-CABG patients predicts long-term mortality.

## 1. Introduction

Coronary artery disease (CAD) is the third greatest cause of mortality in the word. Coronary artery bypass grafting (CABG) surgery is one of the leading therapeutic strategies for patients with significant CAD [1]. CABG surgery reduces manifestations and improves prognosis, but unfavorable cardiovascular events, including cardiac arrhythmias may be observed in post-CABG patients [1, 2, 3]. Importantly, changes in cardiac autonomic regulation are noted in these patients both before and after surgery and may be assessed non-invasively using heart rate variability (HRV) derived from a continuous electrocardiography (ECG) recordings. HRV characterizes oscillations/variations in instantaneous sinus heart rate and has been calculated using various mathematical algorithms since its clinical value was first appreciated in 1963 [4, 5]. At that time, Hon and Lee [6] recognized that fetal distress was preceded by changes in inter-beat intervals recorded on fetal monitoring. Later it was discovered that 24-hr HRV from a Holter recording is a powerful predictor of death following acute myocardial infarction (MI) [7]. This stunning finding brought the potential for Holter-based HRV to add to risk stratification to cardiology. Although the original cut point of the standard deviation of normal-to-normal intervals (SDNN) <50 ms associated with a 3.1 higher adjusted odds of mortality post-MI, has been raised by better care of MI patients, identification of the optimal HRV measures and covariates for risk stratification post-MI is a continuing area of research that is currently benefitting from the use of machine-learning approaches both to develop and test novel HRV measures and to create the optimal models for different subgroups of patients. Since the original 1987 Kleiger *et al*. [7] publication that brought HRV to cardiology, HRV has been the focus of multiple areas of clinical research where measurement has permitted the study of physiological phenomena, disease pathologies, responses to pharmacological and non-pharmacological interventions, as well as continuing to be a tool for risk stratification and outcome prediction.

There are several approaches to the measurement of HRV, virtually all derived from the time series of normal to normal (NN) inter-beat intervals [8, 9, 10, 11]. Time domain methods describe statistical properties of the NN intervals and assess the amount of HRV seen during recordings that may range from 2-min to 24-hr or even more in duration [12]. Time domain HRV parameters can be categorized as: variables that came from evaluations of the NN intervals or instantaneous heart rate, and variables derived from the variations between successive NN intervals.

Frequency domain HRV parameters provide data regarding the difference in heart rate patterns at different underlying frequencies (spectral analysis) and can potentially be linked to physiologic rhythms [6]. Spectral analysis of HRV is generally calculated using a Fast Fourier Transform (FFT) which assumes that the total variance in the heart rate time series can be decomposed into underlying oscillatory components, much like a single note played by an orchestra can be decomposed into its underlying sounds. Spectral components derived from short-term recordings (often 2–5 mins) and long-term recordings (24-hr) differ. The distinction between long-term and short-term recordings is important and often lost when a particular finding about HRV is promoted by investigators. Short-term recordings, usually captured at rest, permit assessment of three main spectral components of HRV: high frequency (HF) power, low frequency (LF) power, and very low frequency (VLF) power [7, 13]. Longer-term recordings generally capture HRV during real life activities, including sleep and permit the estimation of the ultra-low frequency (ULF) power component (oscillations over an every-20-minutes to every-24-hr-period and now, with more advanced technology, even multi day recordings). Also, in order to meet the mathematical assumptions underlying the FFT, that the signal being analyzed can be reproduced by an appropriate combination of underlying, regular sine ways (referred to as stationarity) [13] the VLF, LF and HF components, in a longer recording, are calculated and averaged over shorter time periods. However, classic statistical tests can be employed to check the stability of signals of spectral components [14]. Recordings often include arrhythmic events, ectopic beats, missing data and noise [14]. In general, these are handled by interpolating (splining) the missing NN interval data so that they do not add non-physiologic variation in heart rate and, for research purposes, limits on the percent of missing data for frequency domain analyses are pre-specified. For example, for a 24-hr recording to be accepted as providing a reasonable estimate of the subject’s frequency domain HRV, each 5-min segment is required to have at least 80% NN intervals (i.e., <20% splined) and at least 75% of the 5-min segments are required to have acceptable data quality. Short-term recordings, under laboratory conditions, can be free of noise and analysis periods selected to be sufficiently free of ectopy, but their generalizability for clinical studies is limited, because they are only representative of HRV under very controlled, rather than real-life, conditions and carry a risk of selection bias when only short-term ectopy-free periods are selected for analysis [15].

In addition to statistical, time domain parameters and FFT-based frequency-domain HRV indices, the time series of NN intervals may also be represented by patterns which can be analyzed for HRV based on the geometric attributes of the resulting pattern. The geometric patterns can be fitted to a shape that is mathematically defined and the parameters of this shape can be used. These geometric shapes are classified into pattern-based categories such as linear, elliptical, triangular, which express various classes of HRV. The advantage of such geometrical methods is that they do not rely on the analytical quality of the series of NN intervals [16]. However, such methods do require a significant number of NN intervals to build a geometric model.

Heart rate turbulence (HRT), is not based on NNs but rather on the NN response to a single premature ventricular contraction (PVC), although it requires the presence of at least 5 qualifying PVCs on a recording for a reliable estimate [17]. This relatively novel index of HRV has the following elements: turbulence onset, the direct parasympathetically-mediated reaction of the heart rate to the decrease of cardiac output related to a premature ventricular contraction and turbulence slope, which reflects the slope of the return to baseline heart rate after a premature ventricular contraction and reflects the functional health of baroreflex (blood pressure response).

Nonlinear methods of HRV measurements supply data regarding the dynamics of heart rate, not obvious with conventional methods of HRV analysis [16, 18]. Specifically, nonlinear HRV captures the relative organization *vs.* randomness of the heart rate patterns at different scales. One is approximate entropy (ApEn), which is derived from the logarithmic probability that data points which are like each other will remain close over gradual comparisons. Greater uniformity is related to smaller values of ApEn and abnormality with larger ApEn, in other words, it is a measure of randomness or disorder in a system [19]. Another commonly-used non-linear HRV measure is detrended fluctuation analysis (DFA). DFA has two components: ${\mathrm{DFA}}_{\alpha \,1}$, which estimates the fractal properties (degree of randomness or regularity) of the heart rate over a brief period of 4–11 beats (short-term), and ${\mathrm{DFA}}_{\alpha \,2}$ which estimates the fractal effects of the heart rate over 12–20 beats (long-term) [20]. These fractal-like correlation properties of NN interval dynamics are assessed in short and intermediate time scales over a 24-hour period and reflect overall control of the heart.

In this systematic review, we summarize findings regarding the clinical significance of what is known about HRV before and after CABG.

## 2. Methods

**Fig. 1.**
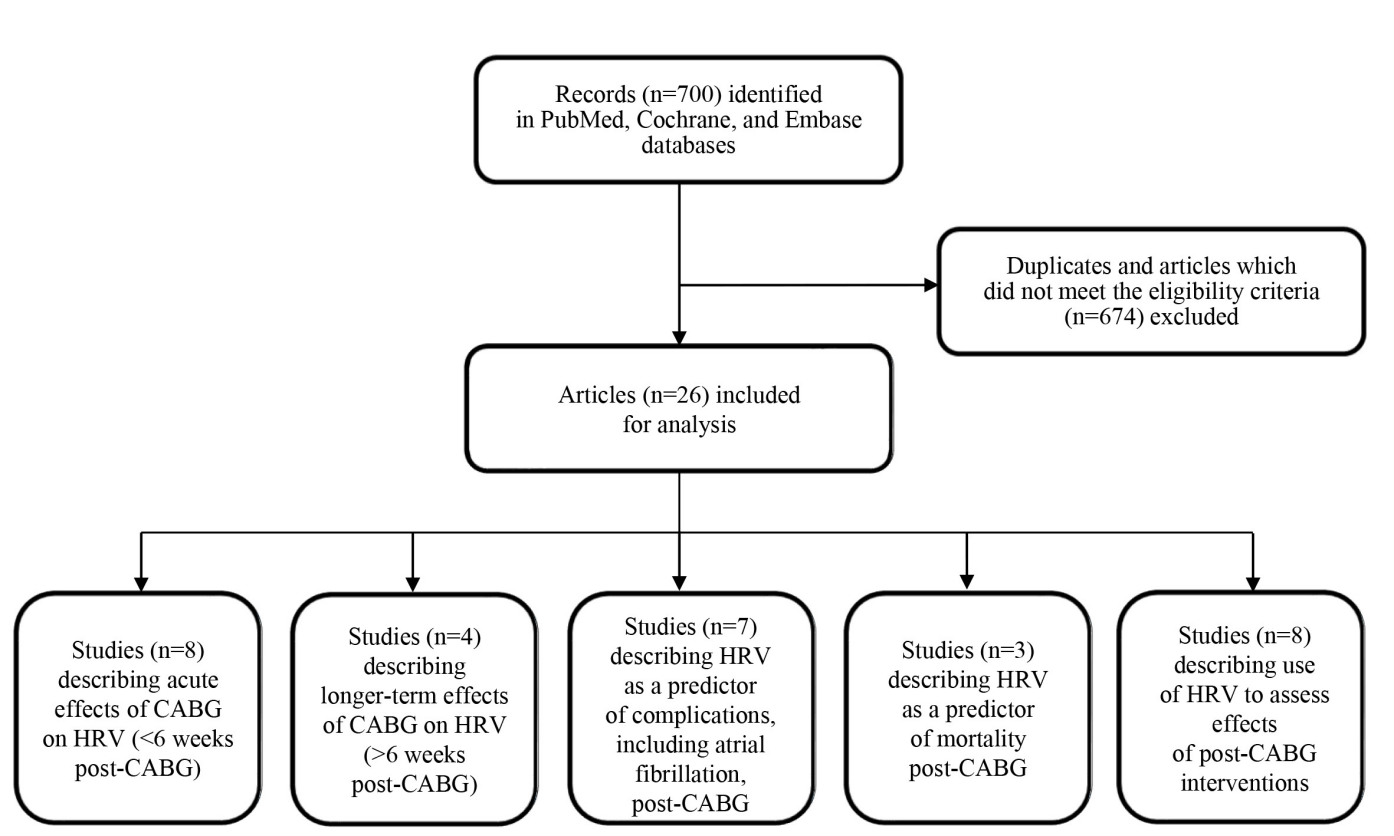
**Flow diagram of research strategy**. Abbreviations: CABG, coronary artery bypass grafting; HRV, heart rate variability.

This literature review was conducted consistent with Preferred Reporting Items for Systematic Reviews and Meta–Analyses (PRISMA) guidelines [21]. The following keywords were used to find relevant papers in PubMed, Cochrane and Embase: “CABG” or “Coronary Artery Bypass Surgery” and “HRV” or “heart rate variability”. Only English language papers found using these keywords and published between the years 1991 and 2022 were checked for importance to the topic. First duplicates were ruled out, and then the initial eligibility evaluation was performed based on titles and abstracts. Afterwards the full texts were checked for eligibility. First, only papers evaluating HRV changes after CABG when comparing with pre-operative HRV status were included in this review. In the second step, papers assessing HRV as a predictor of outcomes after CABG were chosen. Finally, the studies investigating use of HRV to assess effects of post-CABG rehabilitation were included in this analysis. Papers describing potential confounding factors or pre-clinical states and their effect on HRV post-CABG were excluded to preserve focus, but they are summarized briefly in the last paragraphs. The detailed research process is shown in the flow diagram presented in Fig. 1. The latest search was performed on 20 August 2022.

Finally, N = 26 articles were identified for this review. These were categorized into five groups based on their research question (Fig. 1). These five groups were: (1) Acute Effects of CABG on HRV; (2) Longer-term Effects of CABG on HRV; (3) Pre-CABG and post-CABG HRV as predictors of clinical course after CABG; (4) Effects of CABG on the usefulness of HRV as a predictor of mortality after surgery; and (5) Effects of interventions on HRV post-CABG. Additionally, we have described pre-operative clinical conditions and their influence on HRV and confounding factors related to the assessment of the effect of CABG on HRV. Table 1 describes conventional time domain HRV indices, most of which were used in the studies included in the current review, while frequency domain HRV parameters, used in analyzed studies are depicted in Table 2. Table 3 shows examples of HRV nonlinear parameters.

**Table 1.** **Time domain (statistical) parameters of heart rate variability**.

Parameter	Definition
IBI (ms)	Interbeat interval: The interval in ms between successive heartbeats.
RR intervals (ms)	Times between all successive heartbeats.
NN intervals (ms)	Times between normal sinus beats, from which artifacts have been removed.
SDNN (ms)	Standard deviation of the interbeat interval of normal sinus beats.
SDANN (ms)	Standard deviation of the average normal-to-normal intervals calculated over 5-minute intervals.
SDRR (ms)	Standard deviation of RR intervals.
SDNN index (ms)	Mean of the standard deviation of all the normal-to-normal intervals for each 5 min segment of a 24-hr Holter.
pNN50 (%)	Proportion of NN50 divided by the total number of NN (RR) intervals in %.
rMSSD (ms)	Root mean square of successive NN interval differences.
HRV triangular index*	The integral of the density distribution (*i.e*., the number of all NN intervals) divided by the maximum of the density distribution.
TINN (ms)*	Basic width of the RR interval histogram.

*Geometrical measure.

**Table 2.** **Frequency domain measures of heart rate variability**.

Parameter	Definition
ULF power (${\mathrm{ms}}^{2}$)	Ultra-low frequency spectral power (≤0.003 Hz)*.
VLF power (${\mathrm{ms}}^{2}$)	Very–low frequency spectral power (0.0033–0.04 Hz)*.
LF power (${\mathrm{ms}}^{2}$)	Low frequency spectral power (0.04–0.15 Hz)*.
nLF power (nu)	Relative power of the LF band (0.04–0.15 Hz) compared to the combined LF+HF band in normalized units.
LF power (%)	Relative power of the LF band.
HF power (${\mathrm{ms}}^{2}$)	High frequency spectral power (0.15–0.4 Hz)*.
nHF power (nu)	Relative power of the HF band (0.15–0.4 Hz) in normalized units.
HF power (%)	Relative power of the HF band.
LF/HF ratio	Ratio of low frequency power to high frequency power.

*Generally, ln transformed to normalize distributions and permit parametric statistical comparisons.

**Table 3.** **Heart rate variability non-linear parameters**.

Indices	Definition
SD1 (ms)	Standard deviation perpendicular to the line-of-identity in Poincaré plot*.
SD2 (ms)	Standard deviation along the line-of-identity in Poincaré plot*.
SD1/SD2	Ratio between SD1/SD2.
SampEn	Sample entropy assessing time series regularity and complexity.
ApEn	Approximate entropy assessing time series regularity and complexity.
MSE	Multiscale entropy evaluate the complexity of a time series by quantifying its entropy over a range of temporal scales.
${\mathrm{DFA}}_{\alpha \,1}$	Detrended fluctuation analysis evaluating short-term fluctuations.
Shannon entropy	Measures the average information provided by a set of events and proves its uncertainty; quantifying the complexity of physiological signals.
${\mathrm{DFA}}_{\alpha \,2}$	Detrended fluctuation analysis evaluating long-term fluctuations.
D2	Correlation dimension estimates the minimum number of dynamic variables needed to model the underlying system.
SymDyn	Symbolic dynamics reflects changes in cardiac autonomic modulations on short time scales in spite of the considerable reduction of information involved.
β slope	Beta slope is evaluated from the power-law analysis of 24-hour HRV; in healthy subjects, the β index is typically near −1.

* Poincaré plot - a scatter plot where each IBI (IBIn) is plotted against the subsequent IBI (IBIn+1).

## 3. Results and Discussion

### 3.1 Acute Effects of CABG on HRV (<6 Weeks Post-CABG)

Decreased HRV has been shown to be a predictor of death in CAD patients [22]. CABG improves prognosis in this group of patients [23], however little is known about the effect of the procedure on HRV. Although CABG leads to a positive clinical outcome, it is unclear whether CABG surgery simultaneously improves HRV. Published studies of the short-term changes in HRV post-CABG are listed in Table 4 (Ref. [18, 24, 25, 26, 27, 28, 29, 30]). In addition, some of these studies recorded HRV changes beyond 6 weeks post-CABG, these results are discussed in the section following this one.

**Table 4.** **Studies describing acute effects of CABG on HRV (<6 weeks post-CABG)**.

Study	Patients	HRV parameters	Results
Soares *et al*. 2005 [24]	After CABG (N = 13).	Mean RR, rMSSD, SDNN, SDANN, pNN50, TP, LF, HF, LF/HF ratio.	Both time-domain indexes of HRV and TP, and HF power dropped following CABG operation, and recovered 15, 30 or 60 days after CABG. LF and the LF/HF ratio increased early after CABG, then decreased to the pre-CABG levels.
	Control groups (patients with CAD who refused operation [N = 9], and healthy patients [N = 9]).	15-min recordings of paced breathing at 18 cycles/min.	
Ksela *et al*. 2015 [25]	Patients undergoing off-pump CABG (N = 49).	rMSSD, SDNN, SDANN, pNN50, HRV TI, TP, HF, LF, LF/HF ratio.	All observed linear HRV parameters significantly declined 7 days after the surgical procedure, when compared with HRV before surgery.
		24-hr Holter recordings.	
Demirel *et al. *2002 [26]	Patients with CAD undergoing CABG (N = 12).	pNN50, Mean NN, SDANN, SD, rMSSD, SDNN, TP, HF, LF, LF/HF ratio.	All HRV parameters significantly decreased 1 week after CABG.
		24-hr Holter recordings.	
Thanh *et al*. 2022 [27]	Patients with stable CAD (N = 119).	SDNN, rMSSD, SDANN, SDNN index (ASDNN), pNN50, HF, VLF, LF, LF/HF ratio.	Almost all HRV parameters were lower 7 days after the surgery. Only, the LF/HF ratio was unchanged.
		24-hr Holter recordings.	
Bellwon *et al*. 1996 [30]	Patients undergoing CABG (N = 34).	TP, HF, VLF, LF, LF/HF ratio, and %HF.	Before surgery TP, HF, VLF, LF, and %HF dropped after standing up. LF/HF ratio and %LF increased after stand up. After the operation changes described above were no longer observed. Only an increase of HF and %HF in standing position was significant.
		Measured during 10-min periods. Patients were breathing at a controlled rate of 0.25 Hz during the ECG recording.	
Kalisnik *et al*. 2006 [29]	Patients electively admitted for off-pump CABG (N = 42).	TP, LF, HF, nLF, nHF.	HRV measures remained depressed 4 weeks after the procedure.
		10-min ECG recordings.	
Komatsu *et al*. 1997 [28]	N = 10 CABG patients.	Mean RR, LF, HF, LF/HF ratio.	Post-operative changes in cardiac autonomic control with incomplete improvement during the first month after CABG.
		30-min recordings with RR intervals measured with an accuracy of 1 ms.	
Laitio *et al*. 2006 [18]	N = 26 CABG patients (N = 17 complete case included in statistical analysis).	SDNN, ULF, VLF, LF, HF, ${\mathrm{DFA}}_{\alpha \,1}$, ApEn, power law slope (beta).	HF, LF, ULF, VLF dropped after CABG and were decreased six weeks, six months and twelve months after CABG. ${\mathrm{DFA}}_{\alpha \,1}$ was decreased six weeks after operation and increased to the pre-operative values six months after CABG. The beta-slope was stable. The ApEn dropped during the study period.
		24-hr Holter recordings.	

Abbreviations: ApEn, approximate entropy assessing time series regularity and complexity; CABG, coronary artery bypass grafting; CAD, coronary artery disease; ${\mathrm{DFA}}_{\alpha \,1}$, detrended fluctuation analysis evaluating short-term fluctuations; HF, high frequency; hr, hour; HRV, heart rate variability; HRV TI, the integral of the density distribution (i.e., the number of all NN intervals) divided by the maximum of the density distribution; LF, low frequency; pNN50, proportion of NN50 divided by the total number of NN (RR) intervals in %; rMSSD, root mean square of successive NN interval differences; SDNN, standard deviation of the interbeat interval of normal sinus beats; SDANN, standard deviation of the average normal-to-normal intervals calculated over 5-minute intervals; TP, total power; ULF, ultra-low frequency spectral power; VLF, very–low frequency spectral power; RR, times between all successive heartbeats; NN, times between normal sinus beats, from which artifacts have been removed; SD, standard deviation; ASDNN, mean of the standard deviation of all the normal-to-normal intervals for each 5 min segment of a 24-hr Holter. ASDNN is the same as the SDNN index; ECG, electrocardiography.

All studies reported a decrease in traditional HRV parameters immediately after surgery, reaching their lowest value at 3–6 days post-CABG and remaining depressed even 6-weeks follow up after surgery [24, 25, 26, 27, 28, 29]. Thanh* et al*. [27] showed that decreased HRV before surgery was present in 28.6% of patients in 51.8% after seven days, 19.6% after three months, and 12.7% after six months. However, in contrast the finding of Bellwon *et al*. [30] showed that HF and percent of power in the HF band (%HF) increased significantly at 6-weeks post-CABG while in a standing position, but results of this study must be interpreted with caution, because of the brevity of the data- recording time (10 minutes) and because it is not clear that the organization of the HRV signal in the HF power was assessed and that makes it impossible to determine if this increase in HRV reflected a more disorganized cardiac autonomic functioning which has been called “erratic sinus rhythm” [31].

*Summary of results*: HRV decreases acutely after CABG. A study comparing HRV in patients undergoing uncomplicated CABG surgery with patients undergoing non-thoracic vascular surgery found that HRV indices were 40–50% lower in the CABG patients, suggesting that CABG *itself* reduces HRV [26]. Decreases in HRV <6 weeks after surgery may be associated with the acute effects of the operation including: surgical manipulations, prolonged aortic cross-clamping, use of ice slush, ischemia or dysfunction of cardiac ANS regulation post-CABG [32, 33, 34]. Anxiety about the danger and the pain of the surgery are also likely also to contribute to the reduction in HRV [35].

### 3.2 Effect of CABG on HRV (>6 Weeks Post-CABG)

It has now been established, as described in the first section of this review, that HRV decreases acutely after CABG [24, 25, 26, 27, 28, 29]. However, the effect on HRV beyond 6-weeks post-CABG is still unclear. N = 4 studies, listed in Table 5 (Ref. [18, 24, 26, 27]), suggest a recovery of HRV after the initial drop. Demirel *et al*. [26] reported, in N = 12 patients, that standard HRV indices, which decreased after surgery, came back to pre-operative levels after CABG and then improved during the 1-year follow-up. At the 3-year follow-up, all HRV indices recovered to normal values. Soares *et al*. [24], in their study had a CABG group (N = 13) and control groups (patients who refused the operation (N = 9), and healthy subjects (N = 9). HRV was recorded during a 15-min period of paced breathing at 18 cycles/min one day before and 3, 6, 15, 30, 60, and 90 days after surgery. They found that paced-breathing-based HRV reached its lowest value three to six days after CABG and returned to its pre-operative levels at about 2 months post-CABG. Thanh *et al*. [27] demonstrated that, 6 months after surgery, the reperfusion and majority of HRV parameters reflecting injury to the ANS had improved.

**Table 5.** **Studies describing longer-term effects of CABG on HRV (>6 weeks post-CABG)**.

Study	Patients	HRV parameters	Results
Soares *et al*. 2005 [24]	After CABG (N = 13).	rMSSD, Mean RR, SDNN, SDANN, pNN50 (%), TP, LF, HF, LF/HF ratio.	Both time-domain indexes of HRV and TP, and HF power recover by 15, 30 or 60 days after CABG.
	Control groups (patients with CAD who refused operation [N = 9], and healthy patients [N = 9].	15- min recordings of paced breathing at 18 cycles/min.	LF and the LF/HF ratio increased early after CABG, then decreased to the levels before CABG.
Demirel *et al*. 2002 [26]	Patients with CAD undergoing CABG (N = 12).	Mean NN, SDANN, SD, rMSSD, SDNN, pNN50, TP, LF, HF, LF/HF ratio.	HRV indices decreased after CABG, and majority of them recover three months after surgery (only mean NN, rMSSD, and pNN50 were still decreased). LF/HF ratio increased after three months and continued to increase towards pre-operative values six months after CABG.
		24-hr Holter recordings.	
Thanh *et al*. 2022 [27]	Patients with stable CAD (N = 119).	SDANN, SDNN, SDNN index (ASDNN), rMSSD, pNN50, HF, VLF, LF, LF/HF ratio.	HRV indices recover three months after CABG. The percentage of decreased HRV before CABG was 28.6%, 51.8% after seven days, 19.6% after three months, and 12.7% after six months. AS-DNN and SDNN changed the most.
		24-hr Holter recordings.	
Laitio *et al*. 2006 [18]	N = 26 CABG patients (N = 17 complete case included in statistical analysis).	SDNN, ULF, VLF, LF, HF, ${\mathrm{DFA}}_{\alpha \,1}$, ApEn, power law slope (beta).	HF, LF, ULF, VLF power dropped after CABG and were decreased six weeks, six months and twelve months after CABG. ${\mathrm{DFA}}_{\alpha \,1}$ showed the most decrease six weeks after surgery compared to before surgery and increased to the values seen before CABG six months after the operation. Beta-slope was stable. ApEn dropped during the study period.
		24-hr Holter recordings.	

Abbreviations: ApEn, approximate entropy assessing time series regularity and complexity; CABG, coronary artery bypass graft; CAD, coronary artery disease; ${\mathrm{DFA}}_{\alpha \,1}$, detrended fluctuation analysis evaluating short-term fluctuations; HF, high frequency; hr, hour; HRV, heart rate variability; LF, low frequency; pNN50, proportion of NN50 divided by the total number of NN (RR) intervals; rMSSD, root mean square of successive NN interval differences; SDNN, standard deviation of the interbeat interval of normal sinus beats; SDANN, standard deviation of the average normal-to-normal intervals calculated over 5-minute intervals; TP, total power; ULF, ultra-low frequency spectral power; VLF, very–low frequency spectral power; RR, times between all successive heartbeats; ASDNN, mean of the standard deviation of all the normal-to-normal intervals for each 5 min segment of a 24-hr Holter. ASDNN is the same as the SDNN index; NN, times between normal sinus beats, from which artifacts have been removed; SD, standard deviation.

*Summary of results*: The report of a later recovery of HRV to levels like those found in healthy subjects, regardless of the pre-operative status of the subjects and their post-operative clinical condition, suggest that decrease in HRV parameters <6 weeks after surgery is associated with acute effects of CABG and its improvement >6 weeks may be related to success of revascularization, although this too needs to be tested [24].

### 3.3 HRV as a Predictor of Complications including Atrial Fibrillation Post-CABG

Atrial fibrillation (AF) is the most common complication after CABG and is related to a greater risk of heart failure, in-hospital stroke, longer hospital stay and rehospitalization [24, 36, 37, 38, 39]. Importantly, it has been shown that patients at high risk for AF may be recognized using HRV. However, assessment of relationships between HRV and AF risk have been limited to time or frequency domain parameters [40, 41]. Studies (N = 7) describing HRV as a predictor of complications including AF post-CABG are shown in Table 6 (Ref. [40, 42, 43, 44, 45, 46, 47]).

**Table 6.** **Studies describing HRV as a predictor of complications, including atrial fibrillation, post-CABG**.

Study	Patients	HRV parameters	Results
Hogue *et al*. 1998 [40]	Patients with post-operative AF (N = 18) and postoperative control patients without AF (N = 18).	Mean NN, SDNN, pNN50, rMSSD, SD1, SD2, SD1/SD2, ApEn.	HRV indices did not differ between patients and controls.
		Holter recorders for two to three days after CABG.	Decreased ApEn and increased heart rate were related to incident AF.
		HRV analysis in 3 sequential 20-minute intervals preceding the beginning of post-operative AF.	
Tarkiainen *et al*. 2008 [42]	CABG patients (N = 67) of whom N = 48 patients did not have and N = 19 had an AF after CABG.	Mean NN, ${\mathrm{DFA}}_{\alpha \,1}$, SymDyn, ApEn	Preoperative ${\mathrm{DFA}}_{\alpha \,1}$ lower in subjects with AF after CABG than in control group during spontaneous breathing. SymDyn was increased in subjects with incident AF during spontaneous breathing. Increased ${\mathrm{DFA}}_{\alpha \,1}$ during the spontaneous breathing was related to lower risk of incident AF after CABG, while higher SymDyn increased it.
		ECG recordings as follow:	
		(1) 10-min in supine position with spontaneous breathing.	
		(2) 10-min in supine position with controlled breathing.	
		(3) 2-minute in supine position with normal breathing, then 10-min a passive tilt at 70 degree angle.	
Park *et al*. 2014 [43]	CABG patients (N = 113).	HRT onset and power law slope.	Pre-operative abnormal HRT was related to poor short-term and long-term outcomes after operation.
		Ambulatory ECGs recordings 1 to 3 days before CABG.	
Hakala* et al*. 2002 [44]	Elective CABG patients (N = 92).	SDNN, rMSSD, TP, HF, VLF, LF, LF/HF ratio, nLF, nHF.	No measured HRV parameters differed significantly between AF and sinus rhythm patients.
		Continuous ECG recording as follows:	
		(1) 10-min in supine position with spontaneous breathing.	
		(2) 10-min ECG recordings in supine position with controlled breathing.	
		(3) 2-minute in supine position with normal breathing, then 10-min passive tilt at 70 degree angle.	
Chamchad *et al*. 2011 [45]	Patients undergoing off-pump CABG (N = 50).	LF FFT%, HF FFT%, LF/HF, Mean NN, SDNN, rMSSD, NN50, pNN50, SDNN index, TINN, SD1, SD2, SD1/SD2, pPD2, mPD2, entropy.	Decreased LF/HF ratio pre-operatively was related to occurrence of AF after CABG.
		A 10-min ECG recording.	
Laitio *et al*. 2000 [46]	Elective CABG surgery patients (N = 40)	ApEn, SD1, SD2, SDl/SD2, ${\mathrm{DFA}}_{\alpha \,1}$, ${\mathrm{DFA}}_{\alpha \,2}$, SDNN, ULF, VLF, LF, HF.	${\mathrm{DFA}}_{\alpha \,1}$ of the first post-operative 24-hr was the better predictor of prolonged intensive care unit stay. HRV parameters measured before CABG did not predict prolonged intensive care unit stays.
		Pre-operative ECG 12 to 40-hr before CABG in 20 patients.	
		Post-operative ECG after CABG recorded for 48-hr.	
Wu *et al*. 2005 [47]	CABG patients (N = 86)	SDNN, LF, HF, VLF, ${\mathrm{DFA}}_{\alpha 1.}$	${\mathrm{DFA}}_{\alpha \,1}$ is the best parameter to predict post-operative risk.
		24-hr ECG Holter recordings collected before CABG and 24-hr during the 1st post-operative day.	Patients with post-operative prolonged intensive care unit stay, taking inotropes, and having AF had decreased pre-operative and post-operative ${\mathrm{DFA}}_{\alpha \,1}$.

Abbreviations: ApEn, approximate entropy assessing time series regularity and complexity; AF, atrial fibrillation; CABG, coronary artery bypass graft; ${\mathrm{DFA}}_{\alpha \,1}$, detrended fluctuation analysis evaluating short-term fluctuations; ${\mathrm{DFA}}_{\alpha \,2}$, detrended fluctuation analysis evaluating long-term fluctuations; FFT, a fast Fourier transform; HF, high frequency; hr, hour; HRT, heart rate turbulence; HRV, heart rate variability; LF, low frequency; nHF, normalized high frequency; nLF, normalized low frequency; pNN50, proportion of NN50 divided by the total number of NN (RR) intervals; pPD2, correlation dimension expressed as a peak point; rMSSD, root mean square of successive NN interval differences; SD1, standard deviation perpendicular to the line-of-identity in Poincaré plot; SD2, standard deviation along the line-of-identity in Poincaré plot; SDNN, standard deviation of the interbeat interval of normal sinus beats; SymDyn, symbolic dynamics; TP, total power; ULF, ultra-low frequency spectral power; VLF, very–low frequency spectral power; NN, times between normal sinus beats, from which artifacts have been removed; ECG, electrocardiography; TINN, basic width of the RR interval histogram.

Research by Hogue *et al*. [40] based on post-CABG inpatient ECG monitoring and analyzing HRV indices during the hour before the onset of AF, showed that subjects who had AF after surgery had diminished heart rate complexity and more frequent atrial ectopy before the arrhythmia started. They also found that lower ApEn and greater heart rate were independently related to AF. Similarly, another study by Tarkiainen *et al*. [42] found that those who developed AF post-CABG had consistently reduced pre-operative ${\mathrm{DFA}}_{\alpha \,1}$, reflecting a breakdown in fractal R-R interval dynamics and the greater randomness of R-R interval dynamics when breathing normally. These results suggest that the nonlinear HRV parameters evaluated pre-operatively may tell us some extra data about risk assessment of post-operative AF. Interestingly, Park *et al*. [43] showed that pre-operative abnormal heart rate turbulence (HRT) was related to worse both short-term and long-term outcomes after surgery.

In contrast, two other studies found that HRV analysis provided little value in predicting AF after CABG. Hakala* et al*. [44] concluded that the subjects at risk of AF development after surgery could not be recognized with used pre-operative short-term HRV parameters. In their study, assessed HRV indices did not differ between AF and sinus rhythm patients. However, they found that older age and higher body mass index were predictors of AF *after* surgery. Also, Chamchad *et al*. [45] in a study that involved 50 patients who underwent off-pump CABG, concluded that nonlinear HRV parameters may provide minimal value in prediction of AF after cardiac operation. In their study only the LF/HF ratio was decreased pre-operatively in subjects with new onset AF after CABG when compared to subjects without AF. Nevertheless, a more sophisticated real-time analysis of HRV from intensive care unit (ICU) ECG signals, as in the Hogue *et al*. [40] paper cited above may provide a powerful additional tool in identifying at-risk patients and permit timely preventive interventions.

Post-surgical HRV has also been explored as a predictor of length of stay in the ICU. Laitio *et al*. [46] assessed post-operative states of patients throughout their ICU stay. In this study, continuous 24-hr ECG recordings were obtained in 40 adult patients after CABG. They divided the patients into two groups, by length of stay in the ICU: Group A included 33 patients with (ICU stay ≤48 hr) and Group B included 7 patients (ICU stay >48 hr). N = 20 patients had pre-operative (12–40 hr before surgery) continuous 24-hr ECG recordings. Three patients in Group A (short IUC stay) and two in Group B (longer stay) had incidents of AF. As expected, SD of RR intervals and the VLF power, ULF power, LF power, and HF power parameters diminished from pre-operative levels early after CABG. A Poincaré plot analysis, a marker for the organization of beat-to-beat HR patterns, showed *greater *randomness in beat-to-beat heart rates. in the longer ICU stay group Also, they found that ${\mathrm{DFA}}_{\alpha \,1}$ dropped early after operation. Diminished ${\mathrm{DFA}}_{\alpha \,1}$ (24-hrs after surgery) was the best HRV parameter for distinguishing subjects with short or prolonged ICU stays. They also reported that ApEn tended to increase after CABG. These data imply that subjects with less organized fractal-like HR dynamics may have more frequent post-operative complications, including AF or the need to require prolonged ICU care, but that this marker only appears after surgery.

Wu *et al*. [47], in their study, randomized 86 CABG patients into a control and an ischemic preconditioning group (IP). The IP group had induced 2-min ischemia and 3-min reperfusion periods. 24-hr ECG recordings were done both before and after CABG. They found that both standard and nonlinear HRV indices, i.e., ${\mathrm{DFA}}_{\alpha \,1}$ were decreased after CABG both in control and IP group. Diminished pre-operative and post-operative ${\mathrm{DFA}}_{\alpha \,1}$ predicted a greater incidence of post-operative AF and worse post-operative prognosis. However, the reduction in ${\mathrm{DFA}}_{\alpha \,1}$ was less marked in the IP group, suggesting a possible measurable benefit of IP. No other differences were observed in HRV or ectopy parameters between the IP group and controls.

*Summary of results*: From the studies cited above, we can conclude that nonlinear HRV parameters may be better in detection of subtle abnormalities in cardiac autonomic control that traditional HRV indices and may give improved prediction of post-operative MI and post-operative AF in subjects after CABG [33, 34, 42, 46, 47]. Even though the precise mechanism is yet unclear, ischemic preconditioning appears to be effective in improving cardiac performance, decreasing cardiac troponin T levels, decreasing incidence of post-operative arrhythmias and improving post-operative outcome [44, 48, 49]. Although, the clinical use of nonlinear measures of HRV for risk assessment in patients undergoing major cardiac surgery needs to be further confirmed in prospective studies with larger samples, and the use of AI technologies could uncover additional measures, we believe that HRV analysis has shown promise of becoming a clinically useful new prognostic tool for post-operative clinical complications in the assessment of patients scheduled to undergo major cardiac surgeries. Further studies could combine HRV, clinical factors, ECG, peripheral blood biomarkers and/or myocardial properties to improve prediction of adverse clinical outcomes after CABG, as in other cardiovascular diseases [50, 51, 52, 53, 54]. Interestingly, progress in AF detection tools and methods, including wearable and mobile devices may be crucial also for CABG patients [55, 56, 57]. To study the agreement of ECG and photoplethysmographic signals (PPG)-derived HRV, increasingly available on personal devices like smart watches, Chen *et al*. [58] compared their agreement in patients over 1 year after CABG and demonstrated that pulse rate variability measures, might potentially be able to be useful in assessment the ANS regulation of patients after CABG because of the good agreement between the smart watch and ECG-derived for the majority of standard HRV parameters.

### 3.4 HRV as a Predictor of Mortality Post-CABG

Multiple studies have demonstrated that CABG results in an acute decrease in HRV [26, 27, 59, 60]. Lower HRV is also seen in patients undergoing other cardiac surgery, e.g., valve reparation [61]. The probable reasons for the decrease in HRV after surgery include: a surgical intervention on the heart and adjacent structures, extended anesthesia, and cardioplegia. Importantly, as mentioned above, anxiety about the danger and pain of the surgery is likely also to contribute to the reduction in HRV [29]. Studies (N = 3) describing HRV as a predictor of mortality post-CABG are described in Table 7 (Ref. [62, 63, 64]).

**Table 7.** **Studies describing HRV as a predictor of mortality post-CABG**.

Study	Studied group	HRV parameters	Results
Milicevic *et al*. 2004 [62]	N = 175 subjects with decreased HRV after CABG (N = 51) or MI (N = 124).	SDNN.	HRV was lower in CABG subjects than in post-MI group. However, prognosis was better in the operation group than in the post-MI patients.
		24-hr Holter recordings (3 weeks to 3 months after CABG).	
Stein *et al. *2004 [63]	Participants in Cardiac Arrhythmia Suppression Trial (CAST, N = 735).	SDNN, SDANN, SDNN index, TP, VLF, ULF, LF, nLF, HF, nHF, LF/HF ratio, rMSSD, pNN50, pNN625.	Eliminating post-CABG and diabetic subjects improved the relationship of HRV with risk of death.
		24-hr Holter recordings from the CAST (71 ± 120 days after MI).	
Lakusic *et al. *2013 [64]	N = 206 CABG patients.	Mean RR, SDNN, SDNNindex, SDANN, rMSSD, pNN50, TP, VLF, HF, LF, LF/HF ratio.	Subjects with decreased HRV (SDNN) after CABG have worse cardiovascular survival rate than subjects with normal HRV.
		24-hr Holter recordings (3.7 ± 1.4 months after CABG).	

Abbreviations: CABG, coronary artery bypass graft; HF, high frequency; hr, hour; HRV, heart rate variability; LF, low frequency; MI, myocardial infarction; nHF, normalized high frequency; nLF, normalized low frequency; pNN50, proportion of NN50 divided by the total number of NN (RR) intervals; pNN625, percentage of successive RR intervals that differ by more than 6.25%; rMSSD, root mean square of successive NN interval differences; SDNN, standard deviation of the interbeat interval of normal sinus beats; SDANN, standard deviation of the average normal-to-normal intervals calculated over 5-minute intervals; TP, total power; ULF, ultra-low frequency spectral power; VLF, very–low frequency spectral power.

Kalisnik *et al*. [29] concluded that changes in HRV parameters after off-pump CABG are related to adrenergic mobilization which is similar to that seen in on-pump surgeries. Lakusic *et al*. [61] demonstrated that in long-term follow up after CABG surgery HRV parameters were similar both in patients undergoing CABG off-pump and on-pump. Some studies have demonstrated that decreased HRV has no prognostic value in CABG patients, unlike in post-MI patients [62, 63, 65, 66]. Stein *et al*. [63] concluded that excluding post-CABG and diabetes mellitus subjects from HRV analysis strengthens the association between decreased HRV and risk of death. On the other hand, the recent study performed by Lakusic *et al*. [64] compared differences in mortality in patients after CABG with normal *vs.* decreased post-operative HRV. In their study, 24-hr Holter ECGs were obtained on all patients. Unlike some prior reports, results indicated that decreased post-operative SDNN was related to higher risk of death in CABG patients. However, it should be mentioned that patients with diminished HRV after CABG had lower left ventricular ejection fractions, smaller functional capacity and had more bypassed vessels (meaning that a higher degree of CAD, longer operation, and duration of cardioplegia) than patients with normal HRV. All of this may be related to decreased HRV *per se*. Moreover, one of the limitations of this study is that the HRV was not measured before and early after operation (the mean time from the CABG to measuring HRV was 3.7 ± 1.4 months).

*Summary of results*: It has been shown, using both short-term and 24-hr based measures that, in most subjects, HRV declined early after cardiac operation and recovered after a few months of surgery. However, to establish whether diminished post-operative HRV after CABG has prognostic implications, additional studies in a larger, well-characterized sample of subjects are needed. Lakusic *et al*. [64], as do other investigators, emphasized that patients with diminished HRV observed during long term follow-up after CABG should have access to long-term monitoring, diagnostic procedures, and appropriate treatment such as angiotensin-converting enzyme inhibitors (ACE-I), amiodarone and ivabradine [67, 68, 69].

### 3.5 Use of HRV to Assess Effects of Post-CABG Interventions

Patients are exposed to autonomic dysfunction early after their operations, and this makes them more prone to early post-operative complications [26]. Increased parasympathetic tone has been shown to prevent cardiac arrhythmias [70, 71]. Patients with CAD who have decreased parasympathetic nervous activity are at risk of sudden cardiac death, making parasympathetic nervous activity (PNA) a marker with prognostic value [71]. Improvements in measures of parasympathetic functioning have been associated with lower cardiovascular risk [72]. However, sympathetic nervous activity may reflect the severity of heart failure and has prognostic value as well [73]. It has been shown that soon after surgery, sympathetic nervous activity (measured as plasma norepinephrine concentrations) recovers but parasympathetic nervous activity recovery (measured as HF power) lags it [74]. Studies have demonstrated that long-term outpatient cardiac rehabilitation (CR) can modify HRV in a favorable way in patients post-CABG [74, 75, 76, 77, 78]. CR has been shown to have positive effects on subjects with decreased parasympathetic tone before the beginning of rehabilitation post-CABG [78]. Exercise training induces adaptations in the autonomic demand needed during exercise. This adaptation occurs for central as well as peripheral neural pathways [75, 79]. Rehabilitation is aimed at prevention of complications post-CABG, shortening the length of hospital stay and to motivate patients to continue rehabilitation programs in an outpatient setting [80, 81]. Reviewing the scientific evidence today is not possible to sustain that CR as no role after CABG [82]. One study has shown that a short-term CR program, also significantly improves autonomic cardiac regulation at hospital discharge [80]. Amjadian *et al*. [83] demonstrated that both religious practice (Islamic and Qur’an), accompanied by doing homework, and rehabilitation at home with HRV biofeedback therapies, may be useful in subjects after cardiac surgery. It was also shown that various CR programs impacted cardiac autonomic control and length of hospital stay in subjects after surgery [80]. Some studies have shown no reduction in HRV attenuation in subjects after surgery who did not have any CR. Another study attributed this finding in part to prolonged bed rest, warranting early mobilization after CABG [84]. The intensity of the exercise regimen being administered has also been shown to impacts HRV. In one study [84] patients were randomized to an intensive training regimen post-CABG, characterized by supervised CR for one hour, three times a week and a home bicycle program for another three days a week for three months. Results showed that maximal workload capacity increased significantly and persisted one year later. SDNN and SDANN increased significantly, also persisting one year after the CR. A meta-analysis conducted by Kushwaha *et al*. [85] confirmed and reinforced the finding that exercise training improved selected HRV indices, e.g., rMSSD, SDNN, HF power, and LF/HF ratio. Studies (N = 8) describing use of HRV to assess effects of post-CABG interventions are shown in Table 8 (Ref. [74, 75, 76, 77, 78, 80, 81, 84]).

**Table 8.** **Studies describing use of HRV to assess effects of post-CABG interventions**.

Study	Studied group	HRV parameters	Intervention	Results
Jelinek *et al*. 2013 [75]	N = 38 patients, (N = 22 after PCI and N = 16 after CABG).	rMSSD, SDNN, LF, HF, LF/HF ratio.	A 6-week cardiac rehabilitation program both in PCI and CABG patients.	Cardiac rehabilitation, especially in CABG patients improved exercise capacity, cardiorespiratory function and cardiac autonomic regulation.
		A 20-min ECG recording.		
Iellamo *et al*. 2000 [76]	N = 86 patients after CABG randomized into trained (N = 45) control (N = 41) groups.	rMSSD.	Exercise at 85% of maximum heart rate (two daily sessions six times a week for two weeks).	Exercise training increases HRV in CABG patients.
		A 10-min ECG recording.		
Mendes *et al. *2010 [81]	N = 47 patients eligible for CABG (N = 24 EG and N = 23 physiotherapy UCG).	rMSSD, HF, SD1, SD2, ${\mathrm{DFA}}_{\alpha \,1}$, ${\mathrm{DFA}}_{\alpha \,2}$, ApEn, and mean RR, mean HR, LF, LF/HF ratio.	EG had early mobilization with progressive exercises plus usual care.	EG had higher rMSSD, HF, SD1, SD2, ${\mathrm{DFA}}_{\alpha \,2}$, ApEn, and mean RR in comparison to UCG.
			Only respiratory exercises in UCG.	Increased mean HR, LF, and the LF/HF were found in the UCG.
		A 10-min ECG recording in a sitting position.		
Ribeiro *et al*. 2020 [80]	N = 48 CABG patients CG, EMG, or VRG.	Mean RR, rMSSD, SDNN, LF, HF, LF/HF ratio, SD1, SD2, Shannon’s Entropy.	CG—respiratory and metabolic exercises. EMG—cycle ergometer exercises and ambulation.	CG showed a decline in SDNN, rMSSD, and SD1 after CABG. The EMG and VRG had a better cardiac autonomic regulation.
		A 10-min ECG recording.	VRG—cycle ergometer exercises and ambulation with the addition of two Nintendo Wii games during three post-operative days.	
Tygesen *et al*. 2001 [84]	N = 62 patients (43 MI and 19 CABG) randomized to physical training 2 or 6 times per week for three months.	rMSSD, SDNN, SDANN, pNN50, LF, HF, LF/HF ratio.	Bicycle exercise and 24-hr Holter 1, 4 and 12 months after MI or CABG.	SDANN and SDNN increased more in group training 6 times per week.
		24-hr Holter recordings.		HRV changes were more significant in CABG patients.
Bilińska *et al*. 2013 [77]	N = 100 patients after 3 months of CABG (N = 50 EG; N = 50 CG).	SDNN, LF, HF, LF/HF.	Six weeks cycling, 60 min three times per week at 70–80% of HRmax.	Increased SDNN and a tendency towards an increase HF power in exercise group.
		A 24-hr Holter recording.		
Ghardashi-Afousi *et al*. 2018 [78]	N = 42 patients after >6 weeks CABG, LV-HIIT: N = 14; MICT: N = 14; CG: N=14.	SDANN, rMSSD, LF, HF and LF/HF.	LV-HIIT: 10 intervals of 2 min at 85 to 95% of HRmax and separated by 2 min at 50% HRmax for 3/week for 6 weeks. MICT: 40 min running on a treadmill at 70% of HRmax 3/week for 6 weeks.	Higher R-R interval, SDRR, rMSSD, and HF power, and lower LF power and LF/HF ratio in patients after operation in LV-HIIT group.
		A 24-hr Holter recording.		
Takeyama* et al*. 2000 [74]	CABG patients (N = 28; N = 13 exercise group and N = 15 controls).	HF power.	30-min cycling 2/day for 2 weeks in exercise group. Firstly walk 200 m 3/day and then 500 m within 2 weeks in controls.	Increased HF power after three months in exercise group (both at rest and during exercise).
		There are no details about the time of ECG recording (they analyzed 3-min of HRV data).		

Abbreviations: ApEn, approximate entropy assessing time series regularity and complexity; EG, exercise group; CG, control group; UCG, usual care group; EMG, early mobilization group; VRG, virtual reality group; CABG, coronary artery bypass graft; ${\mathrm{DFA}}_{\alpha \,1}$, detrended fluctuation analysis evaluating short-term fluctuations; ${\mathrm{DFA}}_{\alpha \,2}$, detrended fluctuation analysis evaluating long-term fluctuations; HF, high frequency; hr, hour; HRV, heart rate variability; LF, low frequency; MICT, moderate-intensity continuous training; MI, myocardial infarction; LV-HIIT, low-volume high-intensity interval training; PCI, percutaneous coronary intervention; pNN50, proportion of NN50 divided by the total number of NN (RR) intervals; rMSSD, root mean square of successive NN interval differences; SD1, standard deviation perpendicular to the line-of-identity in Poincaré plot; SD2, standard deviation along the line-of-identity in Poincaré plot; SDNN, standard deviation of the interbeat interval of normal sinus beats; SDANN, standard deviation of the average normal-to-normal intervals calculated over 5-minute intervals; ECG, electrocardiography; RR, times between all successive heartbeats.

*Summary of results*: Different kinds of CR improve cardiac autonomic control in patients after CABG. However, in the analyzed studies, there are some methodological discrepancies regarding assessment of HRV, therefore future studies are needed to clarify HRV changes reflecting autonomic adaptation after exercise training in CABG patients and identify risk factors that prevent successful adaptation.

### 3.6 Pre-Operative Clinical Conditions and Their Effect on HRV Post-CABG

CABG improved the quality of life of CAD patients [86]. Nevertheless, about 6–46% of these subjects have been reported to have been exposed to psychological distress and complication related to it [87, 88]. Hallas *et al. * [89] in their study recruited 22 patients undergoing elective CABG for the first time and used the Hospital anxiety and Depression Scale (HAD), the Global Mood Scale (GMS) and the Dispositional Resilience Scale (DRI). Twelve-hour ECG recordings were obtained one week before surgery and two months after CABG, and HRV was assessed. They found that about 40% of subjects were anxious and depressed before their operation, but only 27% after CABG. Depression was the best predictor of reduced HRV assessed both before and after surgery.

*Summary of results*: Depression is related to decreased HRV. It is important to also recognize the role of psychological factors in prognosis after CABG operation and in non-emergent cases, tools to manage this distress might be beneficial to outcome.

### 3.7 Confounding Factors Related to the Evaluation of the Effect of CABG on HRV

As stated in sections 3.1 and 3.2, HRV decreases acutely after CABG and later recovers to pre-operative values. However, the process of undergoing CABG presents many confounding factors that might affect HRV beyond the effect of CABG itself. These confounding factors can be split into three general categories: (1) the perioperative medical management of CABG patients, (2) the process of undergoing cardiac surgery, and (3) characteristics of CABG patients that might affect HRV.

The goal of perioperative medical management of CABG is to minimize perioperative and short-term complications. According to the most recent AHA guidelines [90], medical management should include: insulin infusion, beta blockers and amiodarone. Insulin infusion is indicated to keep blood glucose levels under 180 mg/dL and reduce sternal wound infection. Glycemic control has been shown to improve HRV. In a larger cross-sectional investigation, higher fasting glucose was associated with lower time-domain measures of HRV (RR, rMSSD, SDNN) [91], and in a double-blind randomized controlled trial (RCT) with healthy individuals, manipulation of glucose levels outside the 70–90 mg/dL range caused decreases in SDNN, rMSSD and pNN50 [92].

In addition to insulin infusion, perioperative medical management for CABG also includes preoperative beta-blocker therapy since beta-blockers significantly reduce the risk of AF after surgery [93]. There is general agreement, in the literature, that beta-blockers improve both time-domain and frequency-domain measures of HRV in patients [93]. For example, in a large retrospective study of post-MI patients, use of beta-blockers was related to several increased time-domain HRV parameters (average NN, SDNN, SDANN and SDIDX) [65]. In subjects with decompensated heart failure, beta-blockers were associated with better frequency-domain HRV parameters (total power and ULF) [65, 94]. An experimental study with a rat model of chronic heart failure also reported similar findings, with atenolol and pindolol increasing both LF and HF power [95]. This study revealed augmentation of HRV with the use of beta-blockers supporting their use in patients with heart failure [95, 96]. Another element of perioperative medical management is amiodarone, which also reduces the risk of post-operative AF. There are few studies exploring the impact of amiodarone on HRV, but one experimental study found that amiodarone decreases frequency-domain measures (total power, LF power, LF/HF ratio) in a rabbit model [97].

Finally, traditionally, patients were given aspirin after surgery, to prevent graft occlusion and adverse cardiac events after CABG. The latest AHA guidelines recommend the use of dual antiplatelet therapy (DAPT) up to one year after CABG. However, available evidence is limited to small RCTs and the choice between the use of aspirin *vs.* DAPT remains unclear in CABG [98]. There are two RCTs that characterize the effect of aspirin on HRV, both done on healthy participants: one found that aspirin leads to higher HF and lower LF [99], while another found that aspirin does not have a significant effect on rMSSD [98], although the second may be underpowered. In another RCT, clopidogrel and ticagrelor did not have significant effects on either time- or frequency-domain measures of HRV [100].

Although HRV might be affected by perioperative medical management, it is also affected by the stress of the cardiovascular surgery process itself. For example, anesthetics generally depress both time- and frequency-domain measures of HRV, although different types of anesthetics have different effects. Inhaled anesthetics have been demonstrated to increase HR and decrease HRV (SDNN, LF and HF) [101], while the benzodiazepine remimazolam has been shown to have no effect on LF and HF [102]. Propofol has been shown to decrease HF across two studies, although it is unclear whether it has an effect on LF: one study concludes that it increases LF [102], while another does not find an effect [103]. Another IV anesthetic, thiopental, has similarly been shown to decrease HF and increase LF [104]. The choice of cardioplegia also has an impact on HRV. In an experimental study, warm blood cardioplegia led to significantly higher total power, LF and HF compared to cold crystalloid cardioplegia, although both predictably caused a decrease in HRV [104]. There was also a difference between on-pump versus off-pump CABG: with HRV measured one week and one month. Off-pump CABG led to significantly higher total power, LF and HF compared to on-pump [105], although after a mean of 3.7 months of follow up, there was no difference in HRV by surgery type [106].

There are potentially confounding factors associated with clinical characteristics in CABG patients that need to be considered when assessing changes in HRV parameters. In patients with CAD different HRV changes were observed among subjects with left ventricular ejection fraction (LVEF) over 50%, between 40–50% and below 40% [107]. Differences included normalized HF and VLF as well as LF peak during 5:00–6:00 and 18:00–19:00 [107]. The at-risk group with LVEF <40% had the highest normalized values of these HRV parameters. In another study, positive correlations were observed between SDRR, SDANN, SD, pNN50, LF and HF values and LVEF suggesting that better cardiac function is reflected in better HRV [108]. Traditional linear HRV, multiscale entropy parameters and DFAα_1_ were decreased in subjects with heart failure compared to controls [109]. HRV was also related to the presence (patients with CAD* vs.* controls) and extent (one-, two-, three-vessel CAD *vs.* controls) of CAD as well as dependent on the Gensini score, a scale used for quantifying angiographic atherosclerosis [110]. A number of other cardiovascular risk factors, disorders and comorbidities have been linked to abnormal HRV parameters, including: older age, hypertension, diabetes, peripheral arterial disease, lacunar and atherosclerotic lesions, chronic obstructive pulmonary disease, chronic kidney disease, neuropathy and psychiatric disease [111, 112, 113]. Interestingly, time and frequency domain HRV parameters did not correlate with body mass index in young healthy volunteers [114]. However, in this group which consisted of young healthy adults, waist circumference correlated negatively with SDNN, rMSSD, pNN50, HF normalized units and positively with LF normalized units [114].

*Summary of results*: HRV can be affected by perioperative medical management (insulin infusion, beta-blockers and amiodarone), the cardiovascular surgery itself (anesthetics, cardioplegia) and clinical characteristics of the patients.

## 4. Conclusions

Clinical states such as: heart failure, type 2 diabetes, and depression, adversely affect post-CABG HRV. HRV decreases acutely after CABG but recovers almost completely to its pre-operative value by 6 months after surgery. Traditional time and frequency domain HRV parameters generally fail to predict complications post-CABG, but more abnormal non-linear measures of HRV may identify subgroups of subjects at increased risk of potential complications, including AF, after operation. However, data available currently does not appear to unequivocally support the hypothesis that early HRV assessment in post-CABG patients predicts long-term mortality. Finally, post-CABG cardiac rehabilitation appears to improve exercise capacity and speed up recovery of HRV.

## Supplementary Material

Supplementary material associated with this article can be found, in the online version, at https://doi.org/10.31083/j.rcm2501036.
